# Nanoemulsion Containing Kojic Dipalmitate and Rosehip Oil: A Promising Formulation to Treat Melasma

**DOI:** 10.3390/pharmaceutics15020468

**Published:** 2023-01-31

**Authors:** Júlia Capp Zilles, Larissa Pedron Duarte, Thaís Carine Ruaro, Aline Rigon Zimmer, Irene Clemes Kulkamp-Guerreiro, Renata Vidor Contri

**Affiliations:** 1 Programa de Pós-Graduação em Ciências Farmacêuticas–PPGCF, Universidade Federal do Rio Grande do Sul, Porto Alegre 90610-000, RS, Brazil; 2 Faculdade de Farmácia, Universidade Federal do Rio Grande do Sul, Porto Alegre 90610-000, RS, Brazil

**Keywords:** antioxidant, kojic dipalmitate, nanoemulsion, rosehip oil, tyrosinase inhibition

## Abstract

Melasma is a hard-to-treat hyperpigmentation disorder. Combined incorporation of kojic dipalmitate (KDP), the esterified form of kojic acid, and rosehip oil, an oil with antioxidant and skin-regenerating properties, into nanocarrier systems appears to be a suitable strategy to develop high-performance formulations. A high-energy method (Ultra-Turrax^®^) was used to develop nanoemulsions containing up to 2 mg/mL KDP, 5% rosehip oil, and 7.5% surfactant. Formulations were characterized regarding droplet size, size distribution, pH, density, morphology, KDP content, incorporation efficiency, and stability under different temperature conditions. A scale-up study was conducted. Skin permeation, antioxidant potential, and tyrosinase inhibitory activity were assessed in vitro. Cell viability studies were also performed. Results showed that nanoemulsions containing 1 and 2 mg/mL KDP had incorporation efficiencies greater than 95%, droplet size smaller than 130 nm, suitable size distribution, zeta potential of approximately −10 mV, and good stability over 30 days of refrigerated storage. The nanoemulsion containing 1 mg/mL KDP was chosen for further evaluation because it had lower nanocrystal formation, greater scale-up feasibility and allowed KDP permeation up to the epidermis similarly than observed for 2 mg/mL KDP. This formulation (1 mg/mL KDP) showed antioxidant and depigmenting efficacy, close to that of 1 mM ascorbic acid. No cytotoxicity was observed in formulations concentrations ranging from 0.06% to 1%.

## 1. Introduction

Melasma is a hyperpigmentation disorder that occurs especially on sun-exposed areas, such as the face. This clinical condition is unleashed by an overproduction of melanin in melanocytes, resulting in dark spots arranged in irregular, bilaterally symmetrical, well-defined patterns [1,2]. Treating melasma is challenging and may require aggressive interventions, such as laser therapy, chemical peels, and cryotherapy; the development of novel treatments with whitening properties may become necessary [3].

An alternative to currently available substances is kojic dipalmitate (KDP), the esterified form of kojic acid, which suffers hydrolysis on the skin releasing kojic acid in situ and thereby inhibiting tyrosinase, the enzyme responsible for melanin synthesis [4]. Unlike kojic acid, KDP is considered stable to light, heat, and a wide pH range. However, KDP is a crystalline lipophilic powder difficult to solubilize and incorporate into formulations [5].

Nanoemulsions have small particle sizes, typically below 500 nm, which increases the contact surface. Nanosizing can increase the stability of formulations and, in the case of topical application, enhance the penetration of active ingredients [6,7]. KDP and nanotechnology have been previously associated in a few investigations [5,8,9,10,11]. Lipophilic formulations, such as liposomes, can increase the loading capacity of KDP [8], whereas nanoemulsions can act as efficient carriers that improve the stability [9] and activity [10] of KDP on the skin. Ethosomes have been described as well for also showing stability and deep penetration into the skin [11]. Besides being efficient carriers, nanoemulsions are also known to be a suitable choice of delivery system regarding skin permeation of drugs due to their reduced particle sizes and lipophilic characteristics [6,7], leading to a higher affinity with the stratum corneum and thereby allowing deeper skin penetration/permeation of active substances and, as result, higher efficacy [12]. Moreover, nanoemulsions have lipophilic cores, which are excellent carriers of hydrophobic actives in aqueous media [12]. This study aims to develop an innovative association of KDP with a vegetable oil as a high-activity depigmenting formulation. 

Rosehip oil, extracted from the seeds of *Rosa* aff. *rubiginosa* L., possesses interesting dermatological properties. It is rich in fatty acids, such as linoleic and alpha-linoleic acids, which positively influence the maintenance and repair of skin structure [13,14]. Moreover, rosehip oil contains ascorbic acid, *trans*-retinoic acid, mineral salts, and phenols and shows antioxidant, wound-healing, and regenerative properties [13,14,15]. Application of nanotechnological approaches to rosehip oil was shown to increase the vegetable oil stability and its biological activity [14,16].

In view of the above, it was hypothesized that incorporation of KDP and rosehip oil into a suitable nanoemulsion may provide a stable formulation with high potential in the treatment of melasma. Nanoemulsions were developed and characterized for morphology, physicochemical parameters, in vitro skin permeation, scale-up feasibility, antioxidant activity, tyrosinase inhibitory activity, and toxicity to skin cells.

## 2. Materials and Methods

### 2.1. Materials

KDP was purchased from SM Empreendimentos Farmacêuticos Ltd.a. (São Paulo, Brazil); rosehip oil was purchased from Delaware (Porto Alegre, Brazil); sorbitan oleate was obtained from Croda (Campinas, Brazil); polysorbate 80 was obtained from Labsynth (Diadema, Brazil); tetrahydrofuran (THF) was purchased from Química Moderna (Barueri, Brazil); acetonitrile was obtained from J.T. Baker (State of Mexico, Mexico); methanol and tyrosinase (50 kU) were purchased from Sigma–Aldrich Brasil Ltd.a. (Cotia, Brazil); and 3T3-L1 cells were purchased from the Rio de Janeiro Cell Bank (BCRJ) (Rio de Janeiro, Brazil). All other chemicals or reagents were of analytical grade and used as received. 

### 2.2. Development of Nanoemulsions

An oil-in-water nanoemulsion was developed using rosehip oil. Equal amounts of the surfactants sorbitan oleate and polysorbate 80 were used, according to a previously described method [17]. An aqueous phase consisting of purified water and polysorbate 80 (3.75%) was slowly and constantly poured into an oil phase consisting of KDP (0.1–0.2%), rosehip oil (5%), and sorbitan oleate (3.75%) under heating (60 °C) and magnetic stirring. The final concentration of surfactants was less than 10% to prevent the formation of a microemulsion [18]. A high-energy method with the use of T10 Basic Ultra-Turrax^®^ (IKA, Staufen, Germany) was employed to obtain thermokinetically stable nanoemulsions [19]. Preliminary tests were performed to study the best number of cycles, KDP concentrations, and heating conditions. Six cycles of 10 min at 30,000 rpm, spaced at 5 min intervals, under heating were used. The resulting formulations are referred to hereafter as NERO1KDP (1 mg/mL KDP) and NERO2KDP (2 mg/mL KDP).

For comparison purposes, unloaded nanoemulsions (NERO) were prepared following the same procedures as those described above but without KDP. An unencapsulated KDP dispersion (D1KDP) was also prepared. Briefly, KDP (0.1%) and sorbitan oleate (3.75%) were mixed and heated until solubilization. Then, water and polysorbate 80 (3.75%) were slowly and constantly poured into the mixture, and the formulation was vortexed (VX-18, IONLAB, Araucária, Brazil) for 5 min.

### 2.3. Characterization of Nanoemulsions

Nanoemulsions were characterized for appearance, pH, absolute density, morphology, mean droplet size and size distribution, zeta potential, KDP content, and KDP incorporation efficiency. Measurements were performed in triplicate batches immediately after preparation.

The pH was determined by a potentiometric method (Digimed, São Paulo, Brazil) at room temperature, and density was determined using a glass pycnometer, according to the United States Pharmacopeia [20]. Morphology was analyzed by scanning electron microscopy (Zeiss EVO MA-10, Zeiss). The nanoemulsion was diluted 100 times in purified water. Then, a drop of the dilution was placed on a stub, left to dry at 25 °C, and sputter-coated with gold. (Volume-weighted) average droplet size (*D*_4,3_) and span values were determined by laser diffraction (Malvern^®^ 2000 Mastersizer, Malvern Instruments, Malvern, UK). Average droplet size (*Z*_ave_) and polydispersity index (PDI) were measured by dynamic light scattering (ZEN 3600 Zetasizer^®^ Nano Series, Malvern Instruments, UK) using samples diluted in purified water (1:100). For analyses of both droplet sizes, the refraction index was 1.47. Zeta potential was measured by electrophoretic mobility (Zetasizer^®^ Nano Series, model ZEN 3600, Malvern Instruments, UK) using samples diluted in 10 mM NaCl solution (1:100).

KDP content was determined using a high-performance liquid chromatograph coupled to an ultraviolet detector (HPLC–UV) at 250 nm [21]. Samples were dissolved in THF, acetonitrile, methanol, purified water, and acetic acid (35:30:29:5:1) and kept in an ultrasonic bath for 30 min. The analysis was performed according to Tazesh et al. [21], with modifications. A Shimadzu chromatograph coupled to a UV detector (SPD-10 AV VP, Shimadzu, Kyoto, Japan), and a Restek C18 column (ROC C18, 5 µm, 4.6 × 250 mm, Restek, Bellefonte, PA, USA) were used. The mobile phase consisted of THF, acetonitrile, methanol, purified water, and acetic acid (35:30:29:5:1). The flow rate was set at 1.0 mL/min. KDP content represents the total amount of KDP in the formulation, whereas the incorporation efficiency represents the amount of KDP inside the nanometric droplets.

Incorporation efficiency was evaluated using ultrafiltration–centrifugation (Amicon^®^ 10,000 MW, Millipore, Burlington, MA, USA). Centrifugation was performed at 537× *g* (3 cycles of 10 min each). KDP content was determined in the filtrate by HPLC–UV. Incorporation efficiency (IE) was calculated using Equation (1):

(1) IE =[(Total drug content−Filtrate drug content)/Total drug content]×100

### 2.4. HPLC–UV Method Validation

Specificity, linearity, precision (intra- and interday), and accuracy were evaluated according to International Conference on Harmonization (ICH) guidelines Q2 (R1) for validation of analytical methods [22]. Unloaded nanoparticles were used for specificity assessment. For the linearity test, a standard solution of KDP was prepared in THF, diluted to final concentrations of 1, 5, 10, 20, and 30 µg/mL (in mobile phase), and used to construct three calibration curves on three different days. The average curve was then plotted, and the equation was determined by linear regression. Intraday precision (repeatability) was analyzed in 6 replicate solutions prepared individually at a concentration of 10 µg/mL. Interday (intermediate) precision was determined by taking a total of 9 measurements within the linear interval of the method (10 µg/mL) on 3 different days (3 replicate measurements per day). Accuracy was determined by adding unloaded nanoparticles to KDP dilutions within the linear interval of the method. A total of 9 measurements were performed, with 3 concentrations (low, 8 µg/mL; medium, 10 µg/mL; and high, 12 µg/mL), with 3 replicates at each level.

### 2.5. Stability

Nanoemulsion stability was analyzed by a centrifugation test (30 min at 1006× *g*). When samples did not show signs of phase separation or precipitation, pre-stability tests were performed [22]. Formulations were stored in amber glass flasks at 8, 25, and 40 °C. After 30 days, samples were evaluated for appearance, pH, density, particle size and size distribution, and KDP content. Nanoemulsions were compared with dispersions in terms of KDP content.

For analysis of the presence of KDP crystals in the formulation, KDP content was analyzed in immobilized nanoemulsion samples by HPLC–UV as previously described. Immobilized samples were stored at 25 °C for 30 days and analyzed without being shaken [23]. 

### 2.6. In Vitro Skin Permeation Studies

Skin permeation studies were performed according to da Silva et al. [24], with modifications, using Franz Cells and porcine ear as a membrane (skin thickness 2.17 ± 0.12 mm). The receptor medium was 7.5% polysorbate 80 and 92.5% purified water. The study was conducted at 32 ± 1 °C, with the receptor medium (6 mL) kept under magnetic stirring. An infinite dose regimen was applied. Assays were repeated three times for each sample. First, 1 mL of each sample (1 and 2 mg/mL nanoemulsions) was applied to the membrane in the donor compartment. After 12 h of contact with the membrane, the samples were collected and subjected to HPLC–UV analysis as previously described. The amount of KDP retained on different skin layers was then assessed. The membrane was removed from the cell by using a pair of tweezers. Excess formulation was removed by means of one stripping. The tape-stripping technique (30 tapes) was used to remove the stratum corneum. The tapes were placed in tubes with extraction solution (THF, acetonitrile, methanol, purified water, and acetic acid at a 35:30:29:5:1 ratio). After removal of the stratum corneum, the dermis and epidermis were separated by placing the membrane in water at 60 °C for 45 s. The epidermis was removed with a scalpel, and the dermis was cut into small pieces. The dermis and epidermis were placed separately in tubes containing extraction solution [24]. KDP in its “free” form was not compared with the developed nanoemulsions regarding KDP skin penetration, due to solubility problems.

### 2.7. Scale-Up

This assay was performed to evaluate whether nanoemulsion characteristics were maintained after the scale-up process. Three batches of 350 mL were prepared (10 times higher than the initial batch volume). Nanoemulsions were prepared in the same manner as previously described, at KDP concentrations of 1 and 2 mg/mL, but using a T25 basic Ultra-Turrax^®^ at 13,500 rpm instead of a T10 Basic Ultra-Turrax^®^ (IKA, Germany). Nanoemulsion characteristics (droplet size and distribution, pH, density, KDP content, and incorporation efficiency) were analyzed as previously described.

### 2.8. Antioxidant Activity

Free radical scavenging activity was analyzed by the 2,2-diphenyl-1-picrylhydrazyl (DPPH) assay, as described by Gonçalez et al. [5], with modifications. Briefly, 500 µL of sample (NERO, NERO1KDP, or D1KDP) were added to 3.5 mL of DPPH solution in ethanol (60 µM), kept away from light overnight, and centrifuged for 5 min at 1449× *g*. The absorbance was measured spectrophotometrically (UV-2600 UV–VIS spectrophotometer, Shimadzu, Japan) at 517 nm. Solutions of 1 mg/mL and 0.17 mg/mL (1 mM) ascorbic acid were used as positive controls. Ethanol was used as a negative control. The experiment was performed in triplicate. Free radical scavenging activity was calculated using Equation (2):

(2) $$\mathrm{DPPH}\;\mathrm{inhibition}\;\mathrm{percentage}\;=\left[\left(A_{c}-A_{s}\right)/A_{c}\right]\times 100$$

where *A*_c_ is the absorbance of the negative control, and *A*_s_ is the absorbance of the sample.

### 2.9. Tyrosinase Inhibition Assay

The tyrosinase inhibition assay was conducted as described by Lajis et al. and Berlitz et al., with modifications [25,26]. Briefly, 625 µL of 2 mM l-tyrosine diluted in phosphate buffer (0.1 M, pH 6.8), 313 µL of 0.1 M phosphate buffer (pH 6.8), and 250 µL of sample (NERO, NERO1KDP, or D1KDP) were added to Falcon tubes and incubated for 5 min at 37 °C. Then, 50 µL of phosphate buffer with or without tyrosinase (0.0625 mg/mL from 50 kU tyrosinase) were added to the tubes. After 15 min of incubation (37 °C), dopachrome concentration was determined by absorbance measurement (UV-2600 UV–VIS spectrophotometer, Shimadzu, Japan) at 405 nm. Ascorbic acid solutions (1 and 0.17 mg/mL) were used as positive controls, and phosphate buffer was used as a negative control. The experiment was performed in triplicate. Tyrosinase inhibition percentage was calculated using Equation (3):

(3) Tyrosinase inhibition percentage=[(A−B)/A]×100

where *A* is the difference in absorbance between the control samples with and without tyrosinase, and *B* is the difference in absorbance between test samples with and without tyrosinase. 

### 2.10. Cell Viability Assay

The compatibility of nanoemulsions (NERO and NERO1KDP) with 3T3-L1 mouse embryonic fibroblast cells was evaluated by the 3-(4,5-dimethylthiazol-2-yl)-2,5-diphenyltetrazolium bromide (MTT) assay [27]. Cells were maintained in Dulbecco’s modified Eagle medium (DMEM) supplemented with 10% fetal bovine serum (FBS), 0.5% amphotericin B 250 µg/mL, and a 0.5% solution of penicillin (100 IU/mL) and streptomycin (10 mg/mL). Cells were seeded into 96-well plates at a concentration of 2 × 10^4^ cells/well and incubated overnight at 37 °C in a humid atmosphere containing 5% CO_2_. Then, cells were treated with nanoemulsion concentrations ranging from 0.06% to 1%. Prior to their use in the experiment, the nanoemulsions were sterilized by filtering through 0.22 µm Whatman^®^ syringe filters. The control group was treated with culture medium only (DMEM). After 24 h of treatment, a 0.5 mg/mL MTT solution was added to each well, and plates were incubated for 1 h at 37 °C and 5% CO_2_. Subsequently, the supernatant was discarded, and 100 µL of dimethyl sulfoxide were added to solubilize the formazan crystals. Absorbance was measured using a microplate reader (SpectraMax 190 with ROMTRAmax M2e software version 2.1.35, Molecular Devices) at 570 and 630 nm. Assays were performed in triplicate for each concentration and repeated in three independent experiments. Results are expressed as percentage of viable cells in relation to the control, calculated as follows using Equation (4):

(4) Cell viability (%)=(A/B)×100

where *A* is the mean absorbance of samples, and *B* is the mean absorbance of the control.

### 2.11. Statistical Analysis

Experiments were carried out in triplicate, and results are shown as mean ± standard deviation. Differences between groups were evaluated by one-way analysis of variance (ANOVA) followed by a post-test for multiple comparisons (Tukey’s test) using GraphPad Prism^®^ version 6.01 (San Diego, CA, USA). A value of *p* ≤ 0.05 was considered significant. 

## 3. Results and Discussion

### 3.1. Method Validation

The HPLC–UV method showed good specificity for KDP, being able to detect it separately from other constituents of the formulation, such as rosehip oil and surfactants. The method showed linearity between 1 and 50 µg/mL, with an *R* value of 0.9995. The repeatability (intraday) precision results showed a concentration of 10.77 ± 0.23 µg/mL KDP, considered adequate. The intermediate (interday) precision result was 10.31 ± 0.45 µg/mL KDP, demonstrating good agreement between results of analyses performed on different days. Precision assays showed relative standard deviations of less than 5.0%, in accordance with guidelines [22]. The accuracy test exhibited a mean recovery of 99.78% ± 1.72%.

### 3.2. Formulation Development and Characterization 

Incorporation of 1 and 2 mg/mL KDP at 60 °C resulted in homogeneous formulations, without precipitate. A processing time of 60 min (6 cycles of 10 min) was found to be ideal for droplet size (data not shown). KDP is commonly used at a concentration of 1% in commercial formulations; here, we aimed to incorporate the maximum KDP concentration possible. We were able to incorporate up to 2 mg/mL (0.2%), given that KDP at 3 mg/mL resulted in precipitate formation after centrifugation (data not shown). The efficacy of a substance can be enhanced while lowering toxicity by increasing the amount provided to specific active sites [28]. Nanoemulsions can provide enhanced permeability and skin delivery [18,29]; therefore, KDP concentrations of 1 and 2 mg/mL were chosen for nanoemulsion formulation. 

**Figure 1:**
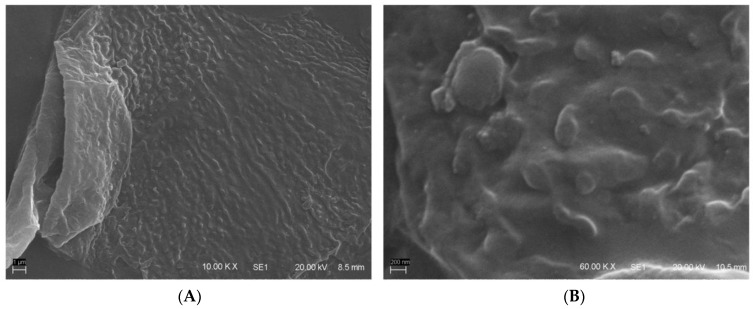
Scanning electron micrographs of 1 mg/mL KDP-loaded nanoemulsion (NERO1KDP) at (**A**) 10,000× and (**B**) 60,000× magnification.

NERO, NERO1KDP, and NERO2KDP were homogeneous, with opaque white color and a bluish brightness. Scanning electron microscopy revealed a spherical morphology, as depicted in Figure 1. Table 1 shows the average droplet size and size distribution, zeta potential, pH, absolute density, KDP content, and incorporation efficiency of nanoemulsion formulations.

Nanoemulsions are defined as colloidal dispersions of 20–400 nm droplets with uniform size distribution [19], in agreement with the formulations described in the present investigation. As shown in Table 1, average droplet size differed according to the analytical technique used. Laser diffraction revealed an average droplet size of 120 nm and dynamic light scattering of 73 nm. Laser diffraction measures the size of a particle by scattering light in a similar way to that of the particles being investigated, thereby being suitable for the measurement of particles with diameters even larger than 1 µm [30]. Therefore, such a method is frequently applied as a primary technique to confirm the monomodal nanometric scale. Dynamic light scattering, on the other hand, determines the hydrodynamic radius of a hypothetical particle scattering light with the same intensity as the investigated particles while in dispersion, and, for this analysis, particles must have a diameter smaller than 1 µm [30,31]. Moreover, the amounts of the samples needed for both techniques varies. Laser diffraction requires larger amounts of the samples while, in DLS, a smaller volume is required [30]. Considering the differences among the techniques, results can differ between both methods [30]. Nevertheless, both analytical techniques revealed appropriate droplet size and particle distribution for nanoemulsions [19]. 

There were no significant differences in particle size, PDI/span value, or zeta potential between loaded (1 or 2 mg/mL KDP) and unloaded formulations. The average droplet size of KDP nanoemulsions was smaller than that of KDP nanoemulsions prepared in previous studies, namely <300 nm [9] and <350 nm [10]. Nanoemulsions prepared with another kojic acid ester, kojic monooleate (KMO), had similar droplet size (approximately 100 nm). Afifah et al. obtained KMO nanoemulsions using 3.19% surfactant (polysorbate 80) and 3.74% oil (9:1 castor oil/lemon essential oil), and they were able to load 10% KMO [32]. Roselan et al. prepared KMO nanoemulsions consisting of 2.7% castor oil and 4% surfactant (polysorbate 80), while also loading 10% KMO [33]. 

The zeta potential of all formulations was −10 mV, indicating that system stability was mainly due to the steric effect [34]. Absolute density was close to 1 g/mL, corresponding to the density of water, and there was no significant difference between formulations. The pH of the skin surface is approximately 5.5, and pH values between 4 and 7 are ideal for topical formulations [35]. Formulations were slightly acidic, being adequate for topical use. The pH values did not differ significantly between the formulations. It is important to emphasize that it would be possible to adjust the pH of formulations after the incorporation of nanoemulsions in cosmetic vehicles, if necessary.

The KDP content of NERO1KDP and NERO2KDP was 101.29% ± 1.62% and 96.67% ± 4.96%, respectively, indicating no drug loss during manufacturing. Incorporation efficiency was 97.79% ± 2.01% and 97.60% ± 1.93% for NERO1KDP and NERO2KDP, respectively, suggesting that there is a high affinity of KDP for rosehip oil, as expected. Nanotechnology allowed the incorporation of up to 0.2% KDP, a lipophilic compound, in an aqueous vehicle, which could facilitate the incorporation of the active into cosmetic formulations.

### 3.3. Stability

**Figure 2:**
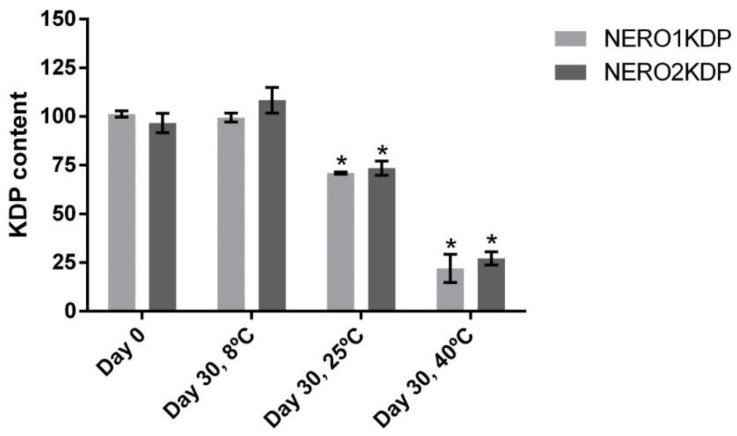
Kojic dipalmitate (KDP) content in 1 mg/mL (NERO1KDP) and 2 mg/mL (NERO2KDP) KDP-loaded nanoemulsions after 0 and 30 days of storage at 8, 25, and 40 °C. An asterisk indicates a significant difference between NERO1KDP or NERO2KDP and the same formulation on day 0 of storage (*p* ≤ 0.05).

NERO1KDP and NERO2KDP did not precipitate after the centrifugation stability test, thereby being considered suitable for pre-stability studies. Pre-stability studies were performed to analyze nanoemulsion stability. Figure 2 shows the KDP content of formulations on days 0 and 30 of storage at different temperatures, namely 8, 25, and 40 °C. Heat affected KDP stability in all formulations, in a way that a decrease in KDP content is enhanced at higher temperatures. Formulations stored at room temperature were found to contain 70% of the initial KDP content, whereas formulations stored at 40 °C contained only 25% of the initial KDP content. Refrigeration at 8 °C promoted KDP stability.

Hydrolysis is the cleavage of labile bonds, such as esters, when in contact with water [36,37]. Hydrolytic degradation can occur in neutral, acid, or alkaline conditions [36]. Since KDP is the dipalmitic ester of kojic acid, and the nanoemulsions are oil droplets dispersed in an aqueous environment, the occurrence of hydrolysis of KDP carbon chains during storage is possible. Such hydrolytic degradation can be accelerated by heat, as storing formulations under elevated temperatures is a method to accelerate hydrolysis reactions [37]. This could explain why refrigerated storage promoted stability regarding KDP content, whereas, under other temperature conditions, there was a decrease in KDP content. Of note, a decrease in KDP content does not necessarily mean loss of activity, given that KDP can be hydrolyzed to kojic acid. Another possibility for the degradation of KDP and its loss of content is the oxidation of the molecule, which can occur in liquid oxidative stress conditions, as mentioned by Tazesh et al. (2021) [21], leading KDP to suffer oxidative degradation, with the suggested mechanism being ring opening [21].

**Figure 3:**
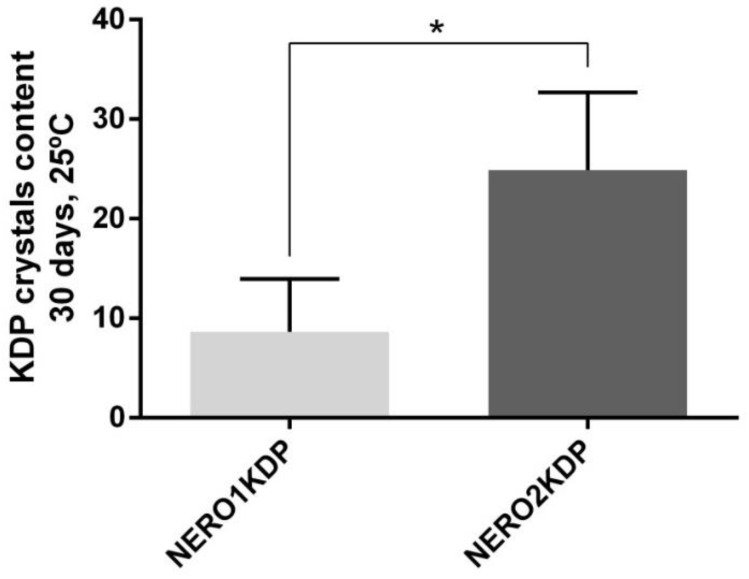
Crystal formation in 1 mg/mL (NERO1KDP) and 2 mg/mL (NERO2KDP) kojic dipalmitate (KDP)-loaded nanoemulsions after 30 days of storage at room temperature (25 °C). An asterisk indicates a significant difference between groups (*p* ≤ 0.05).

Considering the high lipophilicity of KDP, the formation of crystals was also investigated. Figure 3 shows KDP crystal formation after 30 days of storage at room temperature (25 °C). Approximately 10% and 25% KDP crystals were observed in NERO1KDP and NERO2KDP, respectively. NERO1KDP had a significantly lower (*p* ≤ 0.05) crystal content than NERO2KDP.

Regarding the stability of nanotechnology formulations containing kojic acid or its derivatives, droplet size, zeta potential, and PDI were evaluated throughout the duration of the experiments for kojic monooleate nanoemulsions [32,33], kojic acid nanostructured lipid nanocarriers [38], and kojic acid gelatinized core liposomes [39] while submitting the samples to temperatures ranging from 4 to 45 °C. All formulations were considered stable regarding such properties, which is in accordance with our findings, showing that the use of nanotechnology can lead to high stability formulations. Despite its flexibility and tendency to lose shape when in contact with the skin, the nanoemulsions are kinetically stable systems, with promising characteristics for skin application [12]. In 2009, Al-Edresi and Baie studied the formulation and stability of a nanoemulsion (nano-cream) containing KDP [9]. They used large amounts of surfactant (14%), which led to high viscosity of the external phase, and, as a consequence, to a greater physical stability [9]. In the present study, a large amount of surfactant was also used (7.5%), and we were able to develop physically stable nanoemulsions.

NERO, NERO1KDP, and NERO2KDP did not differ significantly in pH, density, particle size, size distribution, or zeta potential (data not shown). The three formulations remained homogeneous and white with a bluish brightness throughout the experiment. The nanometric size of droplets was maintained. Overall, the findings demonstrate that the proposed KDP/rosehip oil nanoemulsions are stable under refrigeration.

### 3.4. In Vitro Permeation Studies

Given that nanoemulsions were developed for topical treatment of melasma, we performed permeation studies to assess whether KDP content influences permeation. Tyrosinase is the enzyme responsible for melanin production. This enzyme is found in melanocytes, which are located in the basal layer of the epidermis [40]; therefore, for melanogenesis inhibition to occur, KDP must reach melanocytes and inhibit tyrosinase.

**Figure 4:**
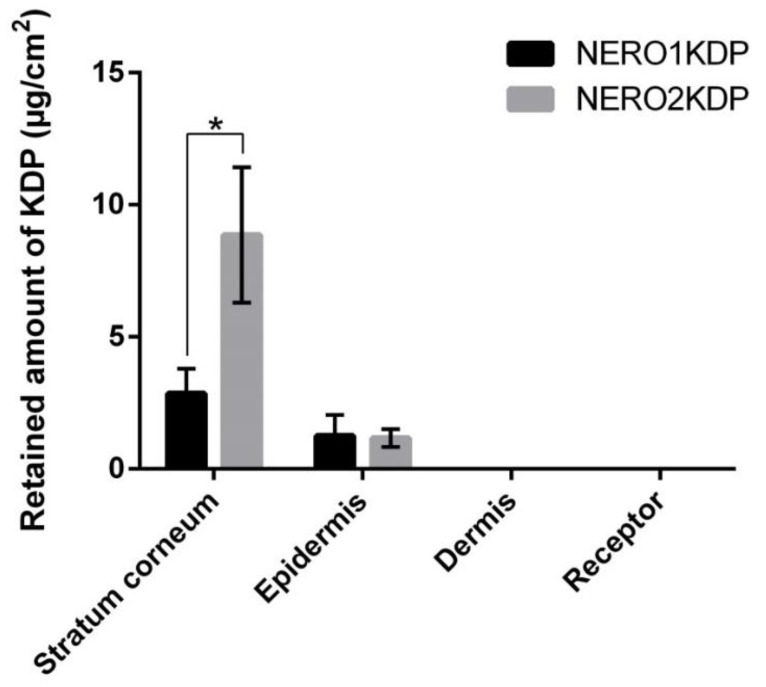
Kojic dipalmitate (KDP) concentrations in receptor medium, dermis, epidermis, and stratum corneum after treatment with 1 mg/mL (NERO1KDP) and 2 mg/mL (NERO2KDP) KDP-loaded nanoemulsions. An asterisk indicate significant differences between formulations for the same skin layer (*p* ≤ 0.05).

Figure 4 shows the amount of KDP from 1 and 2 mg/mL KDP nanoemulsions (NERO1KDP and NERO2KDP, respectively) that permeated the receptor medium after 12 h of contact with the membrane, as well as the amount of the compound found in the stratum corneum (after removal of excess formulation), epidermis, and dermis. KDP did not permeate the full thickness skin after 12 h, as well as it was not possible to quantify the amount of active ingredient in the dermis. These findings are indicative of the safety of the nanoemulsions, as the molecule is expected to act in the viable epidermis, where melanocytes are found [40], and not have systemic action.

After the skin permeation study, KDP is mainly located in the stratum corneum because this is where the formulation is applied, and it is the main barrier of the skin. Although NERO2KDP application resulted in a larger amount of KDP retained in the stratum corneum, this did not reflect in a larger amount of the active ingredient in the epidermis. Approximately 1.2 µg/cm^2^ KDP was found in the epidermis with both NERO1KDP and NERO2KDP treatments, not differing from each other (*p* > 0.05). Given the lack of differences between the formulations, it would be more suitable to use the 1 mg/mL KDP formulation. The skin might have become saturated with KDP in both cases, explaining the lack of difference in epidermal permeation between treatments. According to Guyader et al. (2020) [41], an increase in drug concentration does not mean that an increase in skin penetration will occur, since a saturation of skin flow can happen. It was not possible to quantify the active ingredient in deeper layers of the skin.

### 3.5. Scale-Up

**Figure 5:**
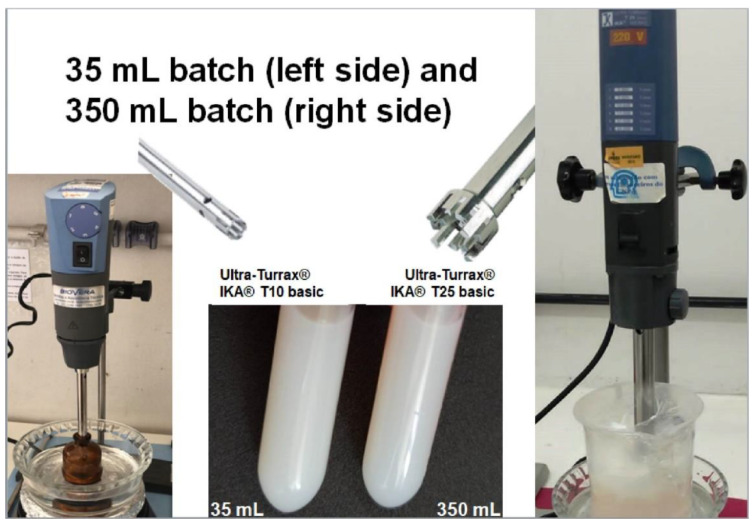
Preparation of 35 mL nanoemulsion batches under heat using a T10 basic Ultra-Turrax^®^ IKA (left) and a process scale-up to 350 mL batches using a T25 basic Ultra-Turrax^®^ IKA (right). The middle panel shows the disperser tools of each piece of equipment and the appearance of formulations.

A method to scale up nanoemulsion production was proposed. Formulations (1 and 2 mg/mL KDP) were produced in batches of 350 mL and showed similar characteristics to formulations obtained in 35 mL batches (Figure 5). There were no significant (*p* > 0.05) changes in droplet size (by dynamic light scattering or laser diffraction), size distribution (span value), or density (data not shown) between laboratory and scaled-up batches. Scaled-up batches did differ, however, with regard to some characteristics: the PDI of NERO2KDP was slightly lower (0.233 ± 0.015), the zeta potential of NERO1KDP was slightly higher (−15.63 ± 0.15 mV), and the pH values of both NERO1KDP and NERO2KDP were slightly lower (6.25 ± 0.07 and 6.34 ± 0.02, respectively). However, such differences did not interfere in nanofeatures or applicability of formulations for topical treatment. Although nanometric dimensions were maintained, only the 350 mL NERO1KDP formulation had a suitable KDP content (97.22% ± 7.93%), and its incorporation efficiency was greater than 95% (102.38% ± 2.06%), being similar to laboratory-scale batches. The KDP content of NERO2KDP was 48.72% ± 6.12%, being only half that of laboratory-scale samples. The loss in KDP content could be attributed to difficulties in phase incorporation, leading to precipitation of the active substance onto the walls of the container. A scale-up of nanoemulsion production may cause inconsistencies in physicochemical characteristics due to errors in multiplication of process parameters or difficulty in handling large volumes [6]. The results showed the potential of NERO1KDP to be scaled up without loss of characteristics. Solubilization and incorporation of KDP are key factors in the scale-up process.

Given the difficulties in scaling up the production of NERO2KDP and the lack of differences between formulations in the amount of KDP in the epidermis and deeper layers of the skin, NERO1KDP (1 mg/mL KDP) is indicated as the most suitable formulation. Moreover, crystal formation was greater in NERO2KDP after 30 days of storage, further corroborating the potential of NERO1KDP.

### 3.6. Antioxidant Activity

**Figure 6:**
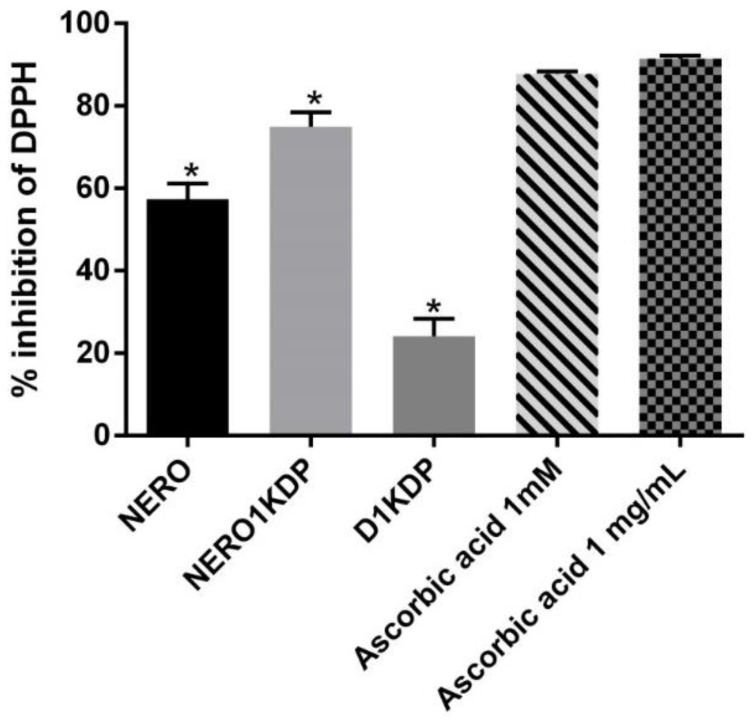
DPPH inhibition percentage of unloaded nanoemulsion (NERO), 1 mg/mL kojic dipalmitate (KDP)-loaded nanoemulsion (NERO1KDP), 1 mg/mL KDP dispersion (D1KDP), and positive controls (1 and 0.17 mg/mL ascorbic acid). One asterisk represents a significant difference from all the other samples (*p* ≤ 0.05).

The DPPH assay, in which the DPPH radical is reduced by antioxidants, was performed to assess the antioxidant activity of the 1 mg/mL KDP nanoemulsion (NERO1KDP) and compare it with that of a 1 mg/mL KDP dispersion and ascorbic acid, a known antioxidant. Results are depicted in Figure 6 and expressed as a DPPH inhibition percentage. Ascorbic acid was used as a positive control at 1 mg/mL (same concentration as KDP in the dispersion and nanoemulsion) and 1 mM (corresponding to 0.17 mg/mL), resulting in a DPPH inhibition of 91.45% ± 0.75% and 87.50% ± 0.38%, respectively, not differing from each other (*p* > 0.05). Park et al. [42] also used ascorbic acid as a positive control and reported an inhibition of 94% at 0.1 mg/mL. This result is similar to the values observed here, although it was achieved with a lower concentration, suggesting that ascorbic acid activity may reach a plateau.

The nanoemulsion loaded with 1 mg/mL KDP had over 70% DPPH inhibition (74.94% ± 3.51%), and, although the value differed significantly (*p* ≤ 0.05) from the positive control, it was numerically similar, demonstrating that KDP nanoemulsion is a promising antioxidant agent. The KDP dispersion also exhibited antioxidant activity, causing 20% DPPH inhibition. The nanoemulsion exhibited higher antioxidant activity than the dispersion, likely because of its nanostructure and association with rosehip oil. Such a result is in accordance with the findings of Khezri et al. (2021), who developed kojic acid nanostructured lipid carriers. The authors observed that nanoparticles had higher antioxidant activity than unencapsulated kojic acid [38]. KDP multiple emulsions were compared with unencapsulated KDP over a period of 28 days by Gonçalez et al. (2015). No differences were observed on day 0, but there was a decrease in antioxidant activity in all formulations over time, with a smaller decrease in nanosystems, attributed to greater KDP stability in multiple emulsions [5].

In the current study, unloaded nanoemulsion exhibited an antioxidant activity of 57.39% ± 3.74%, indicating that rosehip oil does indeed contribute to the antioxidant properties of the formulation. The use of nanotechnology (nanocapsules) in association with rosehip oil has been described, demonstrating that this strategy can increase the stability of vegetable oils and minimize loss in activity [14]. Nanoemulsions of rosehip oil were reported to have antioxidant, anti-inflammatory, and photoprotective activities, showing potential as skin anti-aging and UV protectant agents [16]. Rosehip oil is a good option and has combined effects with KDP on antioxidant activity.

### 3.7. Tyrosinase Inhibition Assay

Tyrosinase is a key enzyme in melanin synthesis, catalyzing important reactions in the melanogenesis pathway. Therefore, it is usually a target of melanogenesis inhibitors and skin-depigmenting agents [43]. Tyrosinase catalyzes the hydroxylation of tyrosine into dihydroxyphenylalanine (DOPA) and then the oxidation of DOPA into dopaquinone, which is spontaneously converted to dopachrome [40]. In the analysis, dopachrome is quantified under UV light at 405 nm [26]. This method assesses whether KDP can inhibit tyrosinase based on the amount of dopachrome formed.

**Figure 7:**
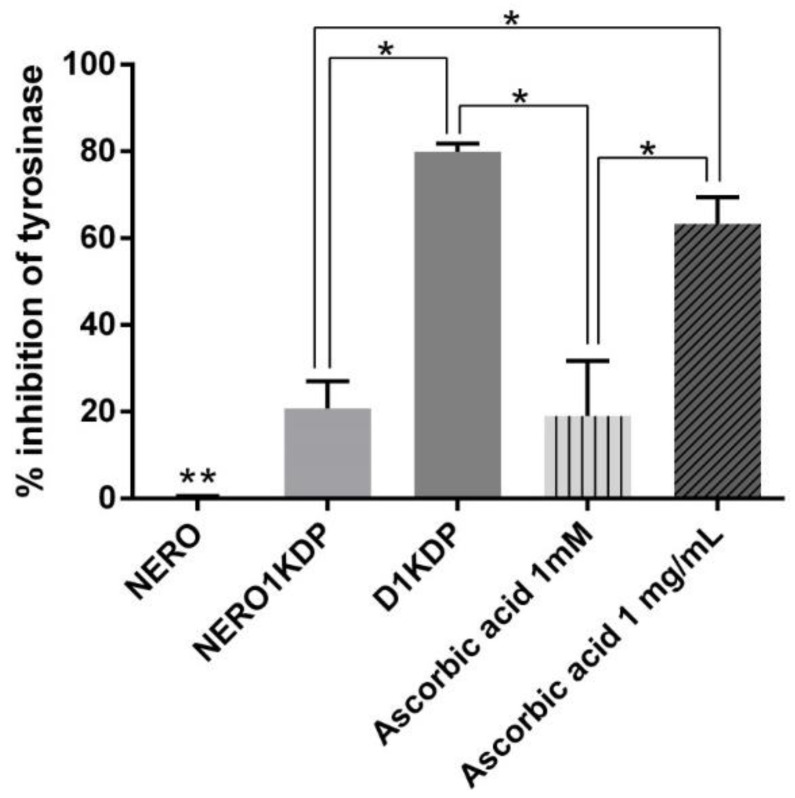
Tyrosinase inhibition percentage of unloaded nanoemulsion (NERO), 1 mg/mL kojic dipalmitate (KDP)-loaded nanoemulsion (NERO1KDP), 1 mg/mL KDP dispersion (D1KDP), and positive controls (1 and 0.17 mg/mL ascorbic acid). One asterisk indicates a significant difference between marked samples. Two asterisks indicate a significant difference from all other samples (*p* ≤ 0.05).

Figure 7 shows the results of NERO1KDP, unloaded nanoemulsion (NERO), 1 mg/mL KDP dispersion (D1KDP), and ascorbic acid (1 mg/mL and 1 mM, corresponding to 0.17 mg/mL) in the tyrosinase inhibition assay. To the best of our knowledge, this is the first study evaluating the tyrosinase inhibitory activity of KDP. NERO1KDP inhibited tyrosinase by approximately 20%, not differing (*p* > 0.05) from 1 mM ascorbic acid, showing the excellent potential of KDP nanoemulsion as a depigmenting agent. Ascorbic acid at 1 mg/mL inhibited tyrosinase by 60%, in agreement with the results of Park et al. [42], who tested the acid at the same concentration. The KDP dispersion (1 mg/mL) did not differ from 1 mg/mL ascorbic acid (*p* > 0.05).

The 1 mg/mL KDP dispersion provided a tyrosinase inhibition of approximately 80%, being greater than that of the 1 mg/mL nanoemulsion (~20%). Roselan (2021) observed that, in a concentration range of 50–500 µg/mL, KMO inhibited tyrosinase activity more efficiently than a KMO nanoemulsion [44], in agreement with our findings. This can be explained by the fact that KDP is retained inside a nanosized oil droplet, impairing contact between the molecule and enzyme. KDP needs to be released from the nanostructure to perform its activity, which did not occur completely given the short experiment time. Of note, under in vivo conditions, the nanoemulsion will likely lose its nanostructure upon skin permeation, releasing KDP from droplets.

Whereas KDP nanoemulsion exhibited tyrosinase inhibitory activity, the unloaded nanoemulsion (NERO) did not. Rosehip oil is rich in ascorbic acid [13], and NERO had antioxidant activity; therefore, it was expected to inhibit tyrosinase, given that antioxidant activity also plays a role in melanin synthesis inhibition [45]. As likely occurred for NERO1KDP, contact between rosehip oil and enzyme was likely impaired by the nanostructure and its controlled-release property, resulting in a more stable formulation.

### 3.8. Cell Viability Assay

**Figure 8:**
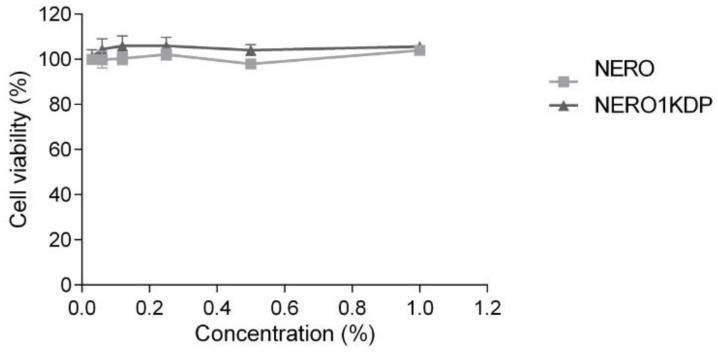
Fibroblast (3T3-L1) cell viability after 24 h of treatment with unloaded nanoemulsion (NERO) and 1 mg/mL kojic dipalmitate (KDP)-loaded nanoemulsion (NERO1KDP) at concentrations ranging from 0.06% to 1%. No significant differences (*p* > 0.05) were observed between samples.

The use of 0.22 µm filters to sterilize formulations and dilutions in cellular media did not alter droplet size or size distribution (data not shown). Near 100% cell viability was observed for all treatments, indicating that neither 1 mg/mL KDP nanoemulsion nor unloaded nanoemulsion were cytotoxic at the nanoemulsions tested concentrations (0.06–1%) (Figure 8). The lack of toxicity of unloaded nanoemulsion demonstrated that the concentration of surfactants used (corresponding to a maximum of 0.075% in wells) was compatible with fibroblasts.

All formulations were found to be safe for fibroblast cells (3T3-L1) at concentrations of up to 1% in each well. The maximum concentration (1% of 1 mg/mL KDP nanoemulsion) corresponded to 16.15 µM KDP. No decrease in cell viability was observed, in agreement with the results for multiple KDP emulsions tested at concentrations of 1 to 18.6 µM in mouse macrophages (J-774 cells) [5]. Overall, the findings demonstrate that the formulation has potential to be safe for cutaneous application.

Previous studies tested the cytotoxicity of KMO nanoemulsions to 3T3 fibroblast cells [32,33]. Afifah et al. found that KMO nanoemulsion and oil had a half-maximal inhibitory concentration (IC_50_) greater than 100 µg/mL, but the survival rate of cells treated with the nanoemulsion was higher, demonstrating the safety of the formulation [32]. In the present study, it was not possible to calculate the IC_50_ because our formulation did not lead to loss of cell viability, even at the maximum concentration tested. At a concentration of 20 µg/mL, KMO nanoemulsion caused a 5% decrease in cell viability [32]. Such differences in cell viability between studies may be attributed to differences in formulations or esterified derivatives. Roselan et al. (2020) obtained IC_50_ values greater than 500 µg/mL for KMO nanoemulsion, also indicating safety of the formulation [33].

It has been shown that 150 nm polymeric particles can penetrate injured skin [46]. Therefore, the KDP nanoemulsions developed in the present study, which have a droplet size of approximately 100 nm, could penetrate the skin, especially when the stratum corneum is injured. This finding underscores the importance of evaluating the safety of formulations.

## 4. Conclusions

Nanotechnology allowed the incorporation of 1 or 2 mg/mL of kojic dipalmitate, a lipophilic skin depigmenting compound, into an aqueous vehicle. The developed nanoemulsions containing KDP and rosehip oil were stable under refrigeration and had suitable nanoscale properties. The nanoemulsion containing 1 mg/mL KDP presented scale-up feasibility, exhibited antioxidant and depigmenting activities, and allowed the active compound to reach the epidermis without permeating to deeper layers of the skin, showing potential for use in cosmetic formulations for melasma treatment. Such nanoemulsion was safe for fibroblast-like cells (3T3-L1) at concentrations up to 1%. 

## Figures and Tables

**Table 1:** Characterization of unloaded nanoemulsion (NERO), 1 mg/mL kojic dipalmitate (KDP)-loaded nanoemulsion (NERO1KDP), and 2 mg/mL KDP-loaded nanoemulsion (NERO2KDP).

Formulation	NERO	NERO1KDP	NERO2KDP
Average size (nm) (laser diffraction)	123 ± 2	117 ± 2	122 ± 5
Span value	0.842 ± 0.009	0.827 ± 0.003	0.918 ± 0.135
Average size (nm) (dynamic light scattering)	72 ± 2	74 ± 1	73 ± 1
PDI	0.281 ± 0.020	0.269 ± 0.004	0.273 ± 0.004
Zeta potential (mV)	−8.57 ± 1.92	−9.44 ± 0.53	−10.24 ± 0.66
Ph	6.7 ± 0.1	6.8 ± 0.1	6.7 ± 0.1
Density (g/mL)	0.9983 ± 0.0002	0.9975 ± 0.0005	0.9977 ± 0.0007
KDP content (%)	-	101.29 ± 1.62	96.67 ± 4.96
Incorporation efficiency (%)	-	97.79 ± 2.01	97.60 ± 1.93

## Data Availability

The data presented in this study are available on request from the corresponding author.
